# Ultrasound-guided platelet-rich plasma injection and multimodality ultrasound examination of peripheral nerve crush injury

**DOI:** 10.1038/s41536-020-00101-3

**Published:** 2020-11-20

**Authors:** Yaqiong Zhu, Zhuang Jin, Jing Wang, Siming Chen, Yongqiang Hu, Ling Ren, Yu Wang, Qing Song, Xiaoqi Tian, Fang Xie, Jiang Peng, Nan Peng, Yukun Luo, Yuexiang Wang

**Affiliations:** 1 Departments of Ultrasound, the first Medical Center of Chinese PLA General Hospital, Beijing, China; 2 Medical College of Nankai University, Tianjin, China; 3 Beijing Key Lab of Regenerative Medicine in Orthopedics, the first Medical Center of Chinese PLA General Hospital, Beijing, China; 4 Key Lab of Musculoskeletal Trauma and War Injuries, the first Medical Center of Chinese PLA General Hospital, Beijing, China; 5 Beijing Key Laboratory of Chronic Heart Failure Precision Medicine, the first Medical Center of Chinese PLA General Hospital, Beijing, China; 6 General Hospital of Northern Theater Command, Liaoning, China; 7 Department of Geriatric Rehabilitation, the second Medical Center of Chinese PLA General Hospital, Beijing, China

**Keywords:** Neuroscience, Regeneration and repair in the nervous system

## Abstract

Ultrasound-guided platelet-rich plasma (PRP) injection is able to make up for the limitations of applying a single growth factor. The goal of this study was to investigate the effects of serial ultrasound-guided PRP injections of the appropriate concentration on the treatment of sciatic nerve crush injury, and explore the value of multimodality ultrasound techniques in evaluating the prognosis of crushed peripheral nerve. In vitro, optimal concentration of PRP (from 150%, 250%, 450%, and 650%) was screened due for its maximal effect on proliferation and neurotrophic function of Schwann cells (SCs). In vivo, ninety rabbits were equally and randomly divided into normal control, model, PRP-2.5×, PRP-4.5×, and PRP-6.5× groups. The neurological function and electrophysiological recovery evaluation, and the comparison of the multimodality ultrasound evaluation with the histological results of sciatic nerve crush injury were performed to investigate the regenerative effects of PRP at different concentrations on the sciatic nerve crush injury. Our results showed that the PRP with a 4.5-fold concentration of whole blood platelets could significantly stimulate the proliferation and secretion of SCs and nerve repair. The changes in stiffness and blood perfusion were positively correlated with the collagen area percentage and VEGF expression in the injured nerve, respectively. Thus, serial ultrasound-guided PRP injections at an appropriate concentration accelerates the recovery of axonal function. Multimodality ultrasound techniques provide a clinical reference for prognosis by allowing the stiffness and microcirculation perfusion of crush-injured peripheral nerves to be quantitatively evaluated.

## Introduction

Peripheral nerve injuries (PNI) are commonly encountered in clinical practice. Although microsurgical approaches contribute substantially to nerve repair, the acceleration of the axonal regeneration and the recovery of nerve function after PNI remains a challenging clinical task^1^.

Recreating a suitable cellular and molecular microenvironment is the focus of functional nerve repair. Various growth factors are among the important constituents of this microenvironment. However, for in vivo approaches, a combined method incorporating fibrin matrix containing growth factor compounds, rather than application of a single growth factor, offers more promising neural tissue repair outcomes^2–4^. In this regard, platelet-rich plasma (PRP) is an attractive alternative that meets the requirements of these combinatorial strategies. It provides a high levels of various natural growth factors and is able to activate different processes, which could make up for the limitations of applying a single growth factor^5^. Once infiltrated around the injured nerve gap as a liquid-to-gel injectable scaffold^6^, tissue fibrinolysis occurs to break down the fibrin, thereby allowing gradual and sustained release of neurotrophic signaling molecules (NGF, BDGF, IGF-1, PDGF, VEGF, HGF) and neurotropic factors (fibrin, fibronectin, and vitronectin)^7,8^. These factors affect many common processes of nerve tissue repair, including regulation of angiogenesis, chemotaxis and cell proliferation, and promotion of extracellular matrix accumulation^9–11^. Moreover, increasing evidence has revealed the beneficial effects of PRP on axon regeneration and neurological recovery in animal and in vitro studies^6,12–15^. However, other studies have reported that PRP has no obvious effect on nerve repair^16^. The inconsistency among results may be due to the differential use of the PRP concentrations and treatment pathways^6,15,17^. Therefore, it is necessary to determine the optimal concentration and treatment approaches of PRP for promoting peripheral nerve regeneration after PNI.

In clinical practice, PRP is often used for local administration, or the injured nerve may be wrapped with PRP membrane during operation, although the latter method may not achieve the therapeutic dose. Therefore, ultrasound-guided serial injection of PRP may be an accurate, widely applicable, novel and safe alternative treatment method for peripheral nerve crush injury.

In addition to guiding the pathway of puncture or thermal ablation treatment, ultrasound has more recently played an increasing complementary role in the clinical evaluation of neuromuscular diseases. Traditionally, nerves are examined using routine high-frequency ultrasound, which provides structural information^18^. Beyond conventional ultrasound, shear-wave elastography (SWE) techniques provide additional information on the elastic properties of tissues^19–21^. Given the histological changes that occur in diseased peripheral nerves, nerve SWE has been explored as a noninvasive approach to evaluate changes in the composition of nerve tissue and as a complementary method to electrodiagnostic studies^22,23^. In addition, contrast-enhanced ultrasonography (CEUS) is a relatively new ultrasound technique that can provide new information about the microcirculation perfusion of the nerve^24,25^. CEUS studies have shown that patients with carpal tunnel syndrome have higher and continuously increasing vascular density and blood flow in the median nerve until at least 3 months after surgery, indicating that blood perfusion of the median nerve may be used in evaluating prognosis^24,25^. However, few studies have used SWE and CEUS to evaluate stiffness and blood perfusion of crushed peripheral nerve or compared the histopathological findings from these approaches to explore the mechanisms of the peripheral nerve regeneration.

The aim of this study was to investigate the effects of serial ultrasound-guided PRP injections of the appropriate concentration on the treatment of sciatic nerve crush injury. The value of multimodality ultrasound techniques (conventional ultrasound, SWE and CEUS) in evaluating the prognosis of crushed peripheral nerves was also explored by comparing the results of multimodality ultrasound and histopathological analysis.

## Results

### Characterization of the PRP

The concentration of platelets in PRP (2130.4 ± 425.8 × 10^3^ platelets/ul) was increased by almost 6.5-fold compared with that in whole blood (320.1 ± 93.1 × 10^3^ platelets/ul). The mean red blood cell (RBC) (7.2 ± 0.9 × 10^9^/ml vs. 1.8 ± 0.4 × 10^9^/ml, *P* < 0.01) and white blood cell (WBC) (11.1 ± 2.9 × 10^6^/ml vs. 2.4 ± 1.0 × 10^6^/ml, *P* < 0.01) counts decreased significantly from whole blood to PRP.

### SC culture experiments

**Fig. 1:**
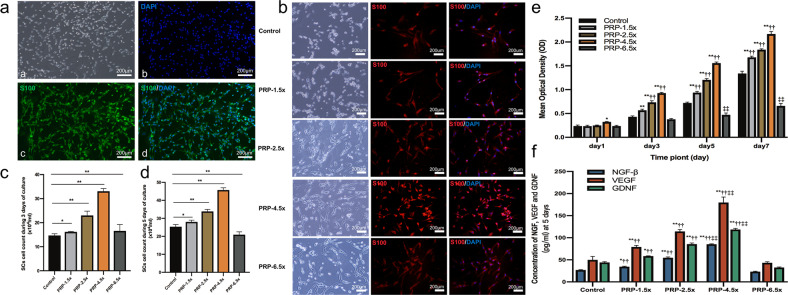
The proliferation and neurotrophin expression of SCs treated with PRP. **a** Immunostaining of harvested SCs. **b** SCs exposed to various PRP concentrations during 5 days of culture. **c**, **d** displayed the SCs count during 3 days and 5 days of culture (*P < 0.05, **P < 0.01). **e** CCK-8 colorimetric assay was performed to evaluate the effect of PRP in various concentration on the proliferation of SCs (**P < 0.01 vs. control group; ^††^*P* < 0.01 vs. PRP-6.5× group; ^‡‡^*P* < 0.05 vs. other five groups). **f** The NGF-β, VEGF and GDNF secreted by SCs that cultured with PRP at different concentrations were measured by ELISA at 5 days of culture (*P < 0.05 **P < 0.01 vs. control group; ^††^*P* < 0.01 vs. PRP-6.5× group; ^‡‡^*P* < 0.05 vs. other five groups). Error bars = SD, *n* = 3. SCs Schwann cells, PRP platelet-rich plasma, CCK-8 Cell Counting Kit-8.

As shown in Fig. 1a, SCs were typically spindle-shaped and often arranged in bundles. Immunostaining with anti-S100, mainly expressed in SCs, showed that the purity of SCs was >95%. The effects of PRP treatment at various concentrations (150%, 250%, 450%, or 650%) on SC viability and proliferation for a given period of time are shown in Fig. 1(b–e). Figure 1b showes gross images and anti-S100-stained immunofluorescence images of treated SCs exposed to different PRP concentrations on day 5. SCs that were not treated with PRP were small and irregularly shaped in most images, whereas SCs were well spread and became more elongated in shape as the dose of PRP increased from 150% to 450% (Fig. 1b). Moreover, the number of SCs increased in a concentration-dependent manner as the PRP dose increased from 150% to 450% (Fig. 1c, d). However, SC proliferation was inhibited by treatment with PRP at a concentration of 650% (Fig. 1c, d).

The CCK-8-based viability assays provided further evidence that PRP with lower than 450% concentration promoted SC proliferation (Fig. 1e). Briefly, the evaluation of cell proliferation at 24 h after treatment revealed no significant difference in the mean optical density (OD) among groups except for in the PRP-4.5× treatment group. However, compared with the control group and the PRP-6.5× treatment group, the proliferation of SCs in the PRP-1.5×, PRP-2.5×, and PRP-4.5× treatment groups increased significantly at day 3, day 5 and day 7 (all *P* < 0.01). Meanwhile, with the exception of the PRP-6.5× group, the results suggested that increases in the concentration of PRP had dose-dependent positive effects on cell proliferation at 3, 5, and 7 days after incubation, and in particularly, 450% concentration of PRP significantly improved cell proliferation and corresponded to the highest average OD value among all groups (all *P* < 0.01). However, the lowest SC proliferation rates among all treatment groups were found for treatment with PRP at the 6.5× concentration on days 5 and day 7 (all *P* < 0.01).

### RPR influenced SC neurotrophic function

ELISA showed that treatment of SCs with 150%, 250%, and 450% PRP induced significant effects on NGF, VEGF, and GDNF secretion within 5 days of culture (Fig. 1f). PRP clearly induced NGF, VEGF, and GDNF secretion in a concentration-dependent manner, and 450% PRP treatment induced the optimal effect (Fig. 1f). However, treatment of SCs with 650% PRP did not significantly elevate the secretion of neurotrophic factors.

### Neurological function evaluation

**Fig. 2:**
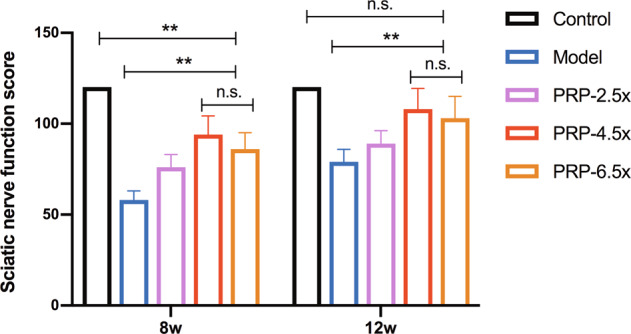
Sciatic nerve function evaluation that rabbits with a sciatic nerve crush injury was repaired with various concentration of PRP treatment after 12 weeks (*n* = 15/group). **P < 0.01, and n.s. not significant. PRP platelet-rich plasma. Error bars = SD, *n* = 3.

Sciatic nerve functional recovery followed the same trend in all injury groups, as nerve function first decreased and then increased with the progression of nerve regeneration (Fig. 2). By 8 weeks, the sciatic nerve function of the PRP-4.5× and PRP-6.5× groups had become distinctly different from that of the model and PRP-2.5× groups (*P* < 0.01), and by 12 weeks, the sciatic nerve function of the PRP-4.5× and PRP-6.5× groups was the most similar to that of the control group. No significant difference in sciatic nerve function was found between the PRP-4.5× group and the PRP-6.5× group.

### Electrophysiological recovery evaluation

**Fig. 3:**
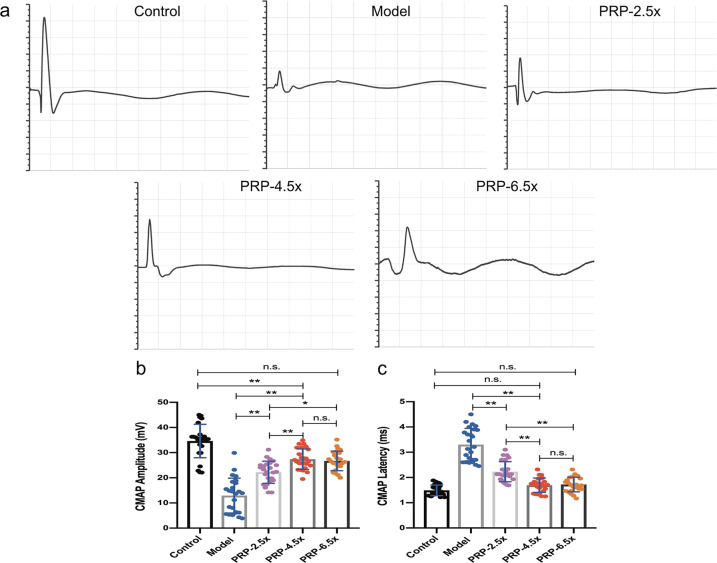
The post-operative electrophysiological data acquired at 12 weeks (*n* = 5/group). **a** Representative CMAP recordings from the damaged side of each group. **b–c** Sciatic CMAP amplitude and CMAP latency comparisons between the injured side of each group. **P < 0.01, and n.s. not significant. Error bars = SD, *n* = 5.

At 12 weeks after surgery, the CMAP amplitude was significantly higher in the PRP-4.5× group than in the PRP-2.5× group and model group (all *P* < 0.01), although, as expected, the CMAP amplitude detected on the treatment side was significantly lower than that recorded from the normal control group (all *P* < 0.01) (Fig. 3a, b). In addition, the PRP-4.5× group exhibited a significantly improved delayed CAMP latency, which was similar to the delayed CAMP latency in the control group (*P* > 0.05) (Fig. 3a, c). In addition, there was no significant difference in the electrophysiological results between the PRP-4.5× group and the PRP-6.5× group (Fig. 3a–c).

### Comparison of the multimodality ultrasound evaluation with the histological results of sciatic nerve crush injury

**Fig. 4:**
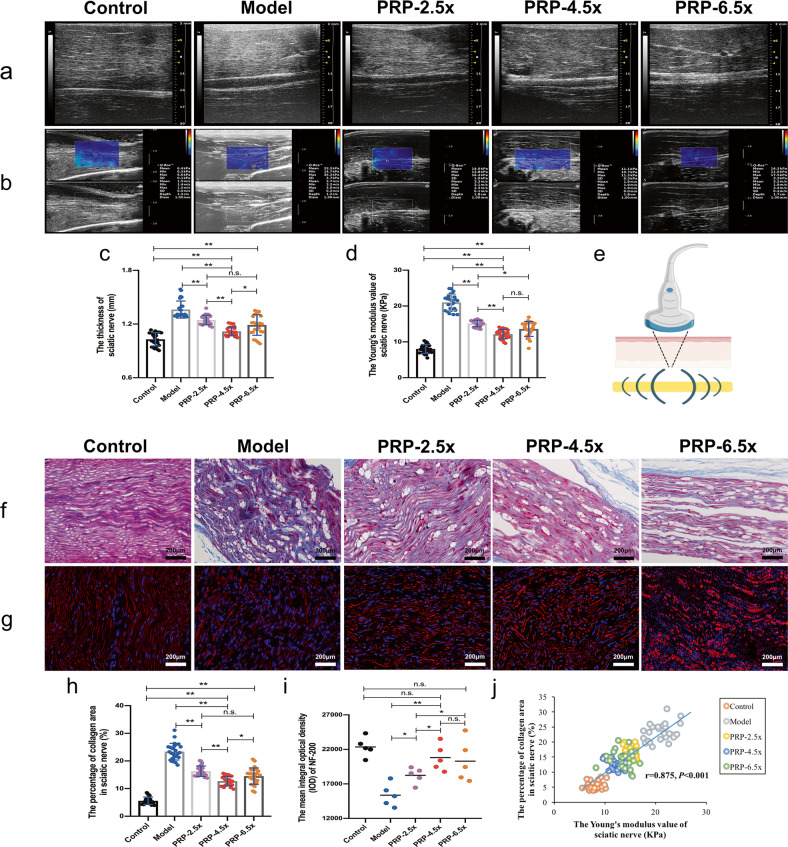
High-frequency ultrasound, shear wave elastography (SWE) and histopathological evaluation of the regenerative nerve at 12 weeks after various concentration of PRP treatment (*n* = 5/group). **a** High-frequency ultrasound images of the sciatic nerve on the surgical side. **b** SWE of the sciatic nerve on the operated side. Statistical analyses were performed by calculating (**c**) the thickness and (**d**) the Young’s modulus value of the treatment area of the sciatic nerve. **e** Illustration of the ultrasound SWE technique used in examination of nerves. Shear wave elastography, where a focused acoustic radiation force is generated by the ultrasound transducer within a region of interest, which leads to the generation of shear waves and tissue deformation. **f** Masson’s trichrome staining at the treatment site of sciatic nerve. **g** Neurofilament 200 staining in longitudinal sections of the regenerative sciatic nerve tissue. Statistical analyses were performed by calculating (**h**) the percentage of collagen area in sciatic nerve, (**i**) the mean IOD of Neurofilament 200 which was used to stain regenerative axons, and (**j**) the correlation between the percentage of collagen area and the Young’s modulus value of sciatic nerve. *P < 0.05, **P < 0.01, and n.s. not significant. Error bars = SD, *n* = 5. SWE shear-wave elastography, PRP platelet-rich plasma, IOD integral optical density.

At 12 weeks, as shown in Fig. 4a, c, the PRP-4.5× group exhibited a significantly improvement in nerve thickness than the PRP-2.5×, PRP-6.5× and model groups, although the nerve thickness was not as great as that in the control group (*P* < 0.01). Staining for NF200, which is expressed in regenerated nerve fibers, revealed a relatively ordered arrangement of axons in the PRP-2.5× group, the PRP-4.5× group and the PRP-6.5× group, while the fiber spacing was more random and asymmetric in the model group (Fig. 4g). According to the IOD analysis, nerve regeneration was significantly promoted in all PRP intervention groups, while the PRP-4.5× and PRP-6.5× groups exhibited better nerve regeneration (Fig. 4i).

The Young’s modulus value of the sciatic nerve in each injury group showed varying degrees of increase at 12 weeks after the operation (Fig. 4b, d, e). Compared with the control group, the PRP-4.5× group and the PRP-6.5× group (the latter tended to be higher than the former) had the smallest increase in nerve elastic modulus, followed by the PRP-2.5× group, while the model group showed the greatest increase in the nerve elastic modulus (Fig. 4b, d). Furthermore, the histological results (percentage of collagen area in the sciatic nerve) were consistent with the change in nerve stiffness in each group (Fig. 4f, h), thus verifying the effectiveness of the SWE assessment. A positive linear correlation was also found between Young’s modulus and the percentage of collagen area in the sciatic nerve (*r* = 0.875, *P* < 0.001) (Fig. 4j).

**Fig. 5:**
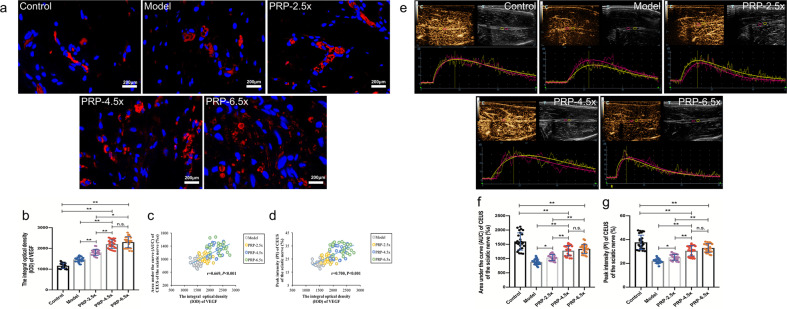
VEGF staining and CEUS detection on the regenerative vessels at 12 weeks post-operation (*n* = 5/group). **a** Vessels were stained with VEGF (red), and nuclei were stained with DAPI (blue) in the control group, model group, PRP-2.5× group, PRP-4.5× group and PRP-6.5× group. **b** The statistical analyses of the mean IOD of VEGF on the operated side. **c** The correlation analysis between AUC of CEUS and the IOD of VEGF. **f** The correlation analysis between the PI of CEUS and the IOD of VEGF. **e** CEUS examination of injured nerve on the operated side. The enhanced time was shown in the horizontal axis and the enhanced intensity was shown in the vertical axis. The time-intensity curves (proximal: pink and distal: yellow) of the CEUS obtained for the control group, model group, PRP-2.5× group, PRP-4.5× group and PRP-6.5× group. **f** Comparisons of the AUC on the CEUS in the operated sciatic nerve among groups. **g** Comparisons of the PI on the CEUS in the operated sciatic nerve among groups. *P < 0.05, **P < 0.01, and n.s. not significant. Error bars = SD, *n* = 5. CEUS contrast-enhanced ultrasonography, PRP platelet-rich plasma, IOD integral optical density, AUC area under the curve, PI peak intensity.

The CEUS of the injured sciatic nerve was quantitatively analyzed at 12 weeks in each group (Fig. 5). The AUC and the PI were obtained by a time-intensity curve, and correlation analyses of the IOD of VEGF expression at the lesion site were performed. The higher AUC and PI indicated better blood perfusion recovery. Both the PI and the AUC were higher in the control group than in the other four operative groups, and blood vessels were arranged regularly and distributed uniformly in the control group (Fig. 5a, e, f, g). Blood perfusion in the PRP-4.5× and PRP-6.5× groups (no significant differences was found between these two groups) was higher than that in the PRP-2.5× and model groups (AUC, PI; all *P* < 0.001), as demonstrated by increased clusters of regenerated microvascular (VEGF expression) in the PRP-4.5× and PRP-6.5× groups (Fig. 5a, b, e, f, g). Moreover, a positive linear correlation between blood perfusion and VEGF expression was observed in the injured groups (the PRP gradient treatment group and the model group) (*r* = 0.669 and *r* = 0.700, all *P* < 0.001) (Fig. 5c, d).

### Histomorphometrical assessment of regenerated nerves

**Fig. 6:**
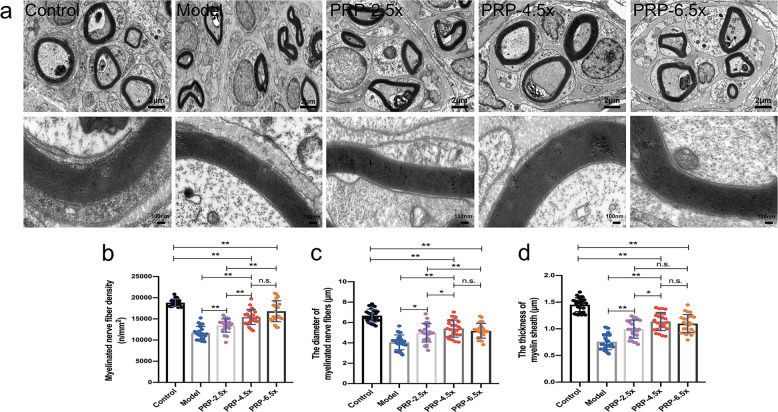
Transmission electron micrography evaluation of regenerated myelinated nerve fibers in the distal portion at 12 weeks post-operation (*n* = 5/group). **a** Transmission electron micrographs of the regenerated nerves in the control group, model group, PRP-2.5× group, PRP-4.5× group and PRP-6.5× group. The histomorphometric analysis was performed by calculating (**b**) the mean myelinated nerve fiber density, (**c**) the mean diameter of the myelinated nerve fiber and (**d**) the mean thickness of the myelin sheath between groups. *P < 0.05, **P < 0.01, and n.s. not significant. Error bars = SD, *n* = 5. PRP platelet-rich plasma.

At 12 weeks after surgery, regenerated myelinated nerve fibers in the distal region of the injury were observed by transmission electron micrography (TEM). The PRP-4.5× and PRP-6.5× groups exhibited significantly higher myelinated nerve fiber density and diameter than those in the PRP-2.5× group the and model group (all *P* < 0.001), but the myelinated nerve fiber density and diameter were still significantly smaller than those in the control group (*P* < 0.001) (Fig. 6a–c). No significant difference was found in the thickness of the myelin sheath among the PRP-4.5×, PRP-6.5× and PRP-2.5 groups (Fig. 6d).

### Triceps surae muscle recovery assessment

**Fig. 7:**
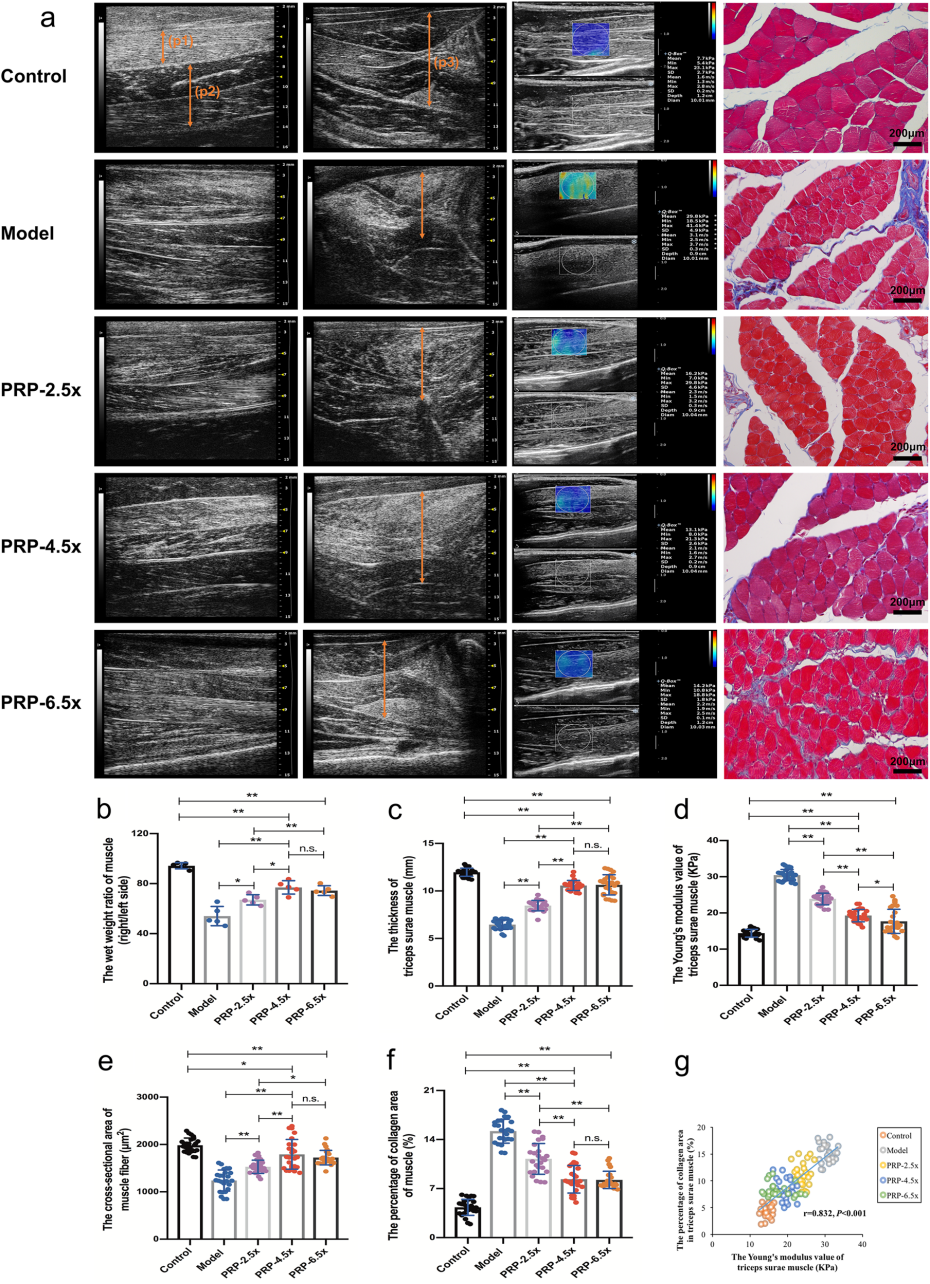
High-frequency ultrasound, shear wave elastography (SWE) and histopathological evaluation of the targeted muscle (*n* = 5/group). **a** Images of the ultrasound and Masson’s trichrome staining. (p1) The gastrocnemius muscle in the longitudinal section. (p2) The soleus muscle in the longitudinal section. (p3) The thickness of the triceps surae muscle in the transverse scan. Statistical analyses were performed by calculating (**b**) the wet weight ratio of triceps surae muscle, (**c**) the thickness and (**d**) the value of Young’s modulus of triceps surae muscle, and (**e**) the cross-sectional area and (**f**) the percentage of collagen area of the muscle fibers. **g** The correlation analysis between the percentage of collagen area and the Young’s modulus value of triceps surae muscle. *P < 0.05, **P < 0.01, and n.s. not significant. Error bars = SD, *n* = 5. SWE shear-wave elastography, PRP platelet-rich plasma.

Twelve weeks after surgery, the targeted muscle in all crushed injury groups showed different degrees of atrophy, which was reflected by increased muscle echogenicity, blurred muscle texture feature, and decreased muscle thickness and muscle wet weight ratio. The PRP-4.5× and PRP-6.5× groups displayed the best recovery of the muscle morphometry (Fig. 7a, b, e, f). The SWE examination indicated that the stiffness of the targeted muscle in the PRP-4.5× group was significantly lower than that in the other crush injury groups, although it was still higher than that in the control group (Fig. 7a, c, g). The PRP-4.5× and PRP-6.5× groups exhibited larger muscle fiber CSA and less collagen production after treatment (Fig. 7d, h, i). Through correlation analysis, the Young’s modulus of the triceps surae muscle in crush injury group was positively linearly correlated with the percentage of collagen area in the targeted muscle (*r* = 0.832, *P* < 0.001) (Fig. 7g).

## Discussion

After traumatic peripheral nerve injury, timely and effective treatment is crucial to prevent the patient from suffering from serious complications and limb dysfunction, and objective and reliable assessment is necessary to provide more reference information for clinical treatment measures.

**Fig. 8:**
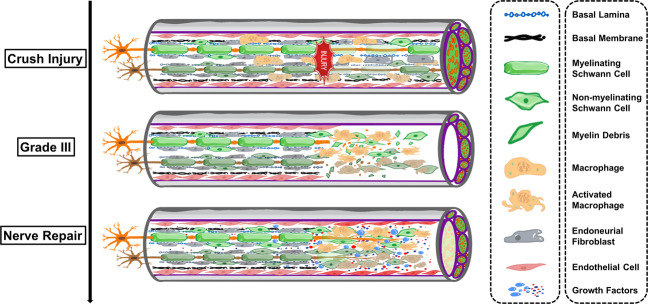
Schematic representation of Sunderland grade III nerve crush injury and nerve regeneration after PRP treatment. Sunderland grade III, axons and the endoneurium were damaged, but not the perineurium. After nerve crush injury, PRP delivers multiple neurotrophic factors (NGF, BDGF, IGF-1, PDGF, VEGF, HGF, etc). This new microenvironment gives way to the survival, proliferation and differentiation of SCs and further promotes the release of neurotrophic factors by SCs. At the same time, promoting vascularization along the fibrin cable precedes axon regeneration.

Previous studies have mostly used single intraoperative PRP treatment that was injected perineurally as a liquid-to-gel injectable scaffold or wrapped around the injured nerve gap as a matrix-like viscous and malleable structure, and conflicting curative results have been reported^16,26^. The inconsistency among results may be due to the differential frequency of PRP treatment and PRP concentrations^6,15,16,26^. Few studies have investigated the therapeutic effect of PRP membrane-wrapped damaged nerves intraoperatively combined with serial ultrasound-guided PRP scaffold injection postoperatively on peripheral nerve crush injury (Fig. 8). In addition, few studies have screened for the optimal concentration of PRP before validation in vivo. Moreover, in terms of assessment, the prognosis of peripheral nerve injury is usually evaluated by clinical manifestations in coinjunction with electrophysiological examination; however, new noninvasive, convenient and reproducible imaging techniques are lacking. Therefore, in this study, we first demonstrated in vitro that PRP with a platelet concentration 4.5 times the platelet concentration of whole blood was the most conducive for promoting the proliferation and secretion of growth factors by SCs, while PRP with a platelet concentration 6.5 times higher than that of whole blood inhibited SC proliferation. Subsequently, we demonstrated in vivo that the PRP-4.5× group could accelerate the regeneration of axon and the myelin sheath and the recovery of target organ function. However, the PRP-6.5× group showed similar nerve repair as the PRP-4.5× group. Furthermore, multimodality ultrasound techniques were used for multidimensionally evaluating the repair status of the nerve according to the degree of swelling, continuity, adhesion degree with surrounding tissues, mechanical properties and microblood perfusion inside the damaged nerve segment. The results showed that conventional US, SWE and CEUS had good reproducibility, and the changes in stiffness and blood perfusion were positively correlated with the collagen area percentage and VEGF expression in the injured nerve, respectively.

Several potential mechanisms may explain how the PRP injections and scaffolds were able to accelerate peripheral nerve regeneration, including by enhancing axonal growth capacity, stimulating angiogenesis, conferring neuroprotection, preventing apoptosis, overcoming the inflammatory microenvironment and dampening denervated target muscle atrophy^27^. Among these possible mechanisms, the promoting effect of SCs (primary structural and functional cells of the peripheral nervous system) plays a critical supportive role in peripheral nerve regeneration^28,29^. In brief, PRP delivers multiple neurotrophic factors (NGF, BDGF, IGF-1, PDGF, VEGF, HGF, etc). This new microenvironment gives way to the survival, proliferation and differentiation of SCs and further promotes the release of neurotrophic factors by SCs^30–32^. At the same time, promoting vascularization along the fibrin cable precedes axon regeneration and is a vital factor in nerve regeneration^33^. Among neurotrophic factors, VEGF is the master switch in the cascade of angiogenesis and, in addition to stimulating angiogenesis, may also act synergistically with TGF-β to reduce inflammation, thus avoiding, or at least diminishing, undesirable consequences such as fibrotic scars^33–35^. Moreover, PRP contains fibrin and fibronectin. Growth factors within the fibrin matrix that are gradually released also include IGF-1, BDNF, NGF, and PDGF, etc., which induce powerful SC proliferation, whereas fibrin and fibronectin simultaneously promote SC migration and homing, increase the myelination of newly sprouted axons, and promote functional recovery of denervated muscles^15,36,37^. As a consequence, it is reasonable to speculate that neurotropic factors and fibrin matrix within the PRP may play a positive role in the phenotype of SCs, which are the master cells of the nerve regeneration process. Based on these mechanisms and advantages, PRP may have broad application prospects in cell therapy and tissue engineering.

PRP concentration was one of the primary prognostic factors. The in vitro experiment performed in this study revealed that the number of SCs increases in a concentration-dependent manner. However, PRP with a 4.5-fold concentration of whole blood platelets, instead of a 6.5-fold concentration of whole blood platelets, could significantly stimulate the proliferation and secretion of SCs, suggesting that <450% PRP was the threshold for stimulating this beneficial effect. According to the CCK-8 assay, the 450% PRP-treated group showed the first signs of incremental effects on SC proliferation on day 1. After that (on days 3, 5, and 7), significant increases in cell proliferation were observed in the 450%, 250%, and 150% PRP-treated groups relative to the control group, and <450% PRP was the threshold value to stimulate the proliferation of SCs. The rapid proliferation and the optimal viability of SCs provide bioactive substrates for axon migration and release molecules that regulate axonal out-growth, thereby accelerating the regeneration of injured peripheral nerves^38^. To investigate the secretion function of SCs treated with various concentrations of PRP after their proliferation, neural factors were also evaluated at 5 days of culture. ELISA analysis showed that the secretion of protein level of NGF, VEGF and GDNF protein, which exert biological functions in promoting the survival and maintenance of peripheral neurons^39^, were significantly increased in the 450%, 250%, and 150% PRP groups. The report of Zheng et al.^15^ was consistent with our finding that showed PRP at the optimal concentration (20% PRP group) had positive effects on SC proliferation and clearly upregulated the expression and induced the secretion of NGF and GDNF in a concentration-dependent manner. The in vivo experiment demonstrated that PRP with a platelet concentration of 4.5-fold and 6.5-fold that of the whole blood was superior to other PRP platelet concentrations in the treatment of sciatic nerve crush injury. At 8 weeks, obvious nerve function recovery began to emerge in the PRP-4.5× group and the PRP-6.5× group, and the difference in recovery became clearly visible in comparison with the model and PRP-2.5× groups; at 12 weeks, nerve function in the PRP-4.5× and PRP-6.5× groups was closest to that of the control group. Similarly, at 12 weeks after treatment, the increase in the density and diameter of myelinated nerve fibers, the increase in myelin sheath thickness, and the recovery of target muscles were the most significant in the PRP-4.5× and PRP-6.5× groups. Therefore, according to our results, the PRP-4.5× group and the PRP-6.5× group showed opposite experimental results in vitro. In vivo, however, the results of the two groups were similar. This may be because the medium containing the same concentration of PRP has different effects on cells in vitro and in vivo due to various factors, such as different microenvironments and different concentrations of SCs. These findings may also indicate that higher platelet concentration does not necessary lead to better the regeneration of injured peripheral nerves, supporting to some extent the results of the in vitro experiments

The duration of action of PRP is another major factor affecting prognosis. A study demonstrated that PRP exerts its effects for approximately 2–4 weeks^40^, and thus, we adopted a method by which injections were administered every two weeks for a total of 3 injections to supplement the treatment and extend the activation time.

Recently, many animal experiments have also demonstrated the beneficial effects of PRP as a filler, a fibrin matrix, or both on peripheral nerve regeneration^6,34,36,41,42^. Cho et al.^13^ and Wu et al.^34^ reported that neuroprotective and antifibrotic beneficial effects of PRP injection into the corpus cavernosum in a bilateral cavernous nerve injury rat model and PRP application in a facial nerve suture in a guinea pig model. Borselli et al.^3^ used an injectable scaffold loaded with VEGF and IGF-1 to accelerate the regeneration of damaged neuromuscular junctions and also observed enhanced angiogenesis. In a rat model, it has been reported that repairing a 10 mm sciatic nerve gap with a vein graft filled with PRP induced more significant early neovascularization than treating the sciatic nerve gap with neural autografts alone^41^. Sánchez et al.^6^ reported an earlier electrophysiological response, a higher axonal density, and lower muscular atrophy after applying PRP as both a filler for damaged nerve and as a scaffold for wrapping crushed nerves of sheep. Another recovery burden of nerve repair is scarring, which has been reported to be minimized by PRP as an adjuvant therapy for the repair of injured sciatic nerves^14^. In addition, there were restricted case reports in a clinical study of ultrasound-guided PRP injection for treating common peroneal nerve trauma, digital nerve crush injury and carpal tunnel syndrome^43–45^. However, few systematic studies that have simultaneously incorporated both the selection of PRP concentration in vitro and verification of the optimal concentration in vivo have been conducted, and the effects have not been studied using serial ultrasound-guided injection therapy. Therefore, this study not only screened for the optimal PRP concentration in vitro and used a traditional intraoperative PRP membrane to encapsulate the injured nerve but also adopted a novel postoperative therapy of serial ultrasound-guided PRP injections to prolong the time of action. The methods and results of this study may be a valuable reference for the clinical treatment of peripheral nerve crush injury.

Autologous PRP can be prepared from autologous peripheral blood through a simple, safe and economic procedure, which may be suitable for nerve tissue reconstruction therapy without inducing a risk of immune rejection or infection. We specifically chose to use autologous PRP for the treatments in this study in order to obtain preclinical research results. Although it is still unclear whether white blood cells in PRP are useful for nerve regeneration, tissue healing may be hindered by the production of bioactive catabolic cytokines^46^. Therefore, PRP with a relatively low leukocyte concentration was extracted in this study.

Ultrasonography is the preferred imaging technique for investigating nerve diseases owing to its widespread application, relatively low cost, and its potential for use in combination with nerve conduction studies. By using routine high-frequency ultrasound, the anatomical localization, edema, continuity, echogenicity and mobility of the injured nerve can be assessed. In this study, the PRP-4.5× group exhibited significantly improvement in nerve thickness than the PRP-6.5× group, but there were no significant differences in the electrophysiological results or histological examination results of axonal regeneration between the two groups. The findings may demonstrate that routine high-frequency ultrasound examination is insufficient to adequately evaluate nerve regeneration. Hence, conventional ultrasound examination limits the exploration of the stiffness and intraneural microcirculation of the injured nerve, although identifying changes in these parameters is the primary aim in evaluating the pathological process of nerve crush injury. Therefore, detection of the injured nerve by SWE and CEUS is essential.

In this study, SWE was used to detect and quantitatively analyze the stiffness of the injured sciatic nerve and its innervated target muscle in each group. Histological evaluation confirmed that Young’s modulus value was positively linearly correlated with the percentage of collagen area in the sciatic nerve and triceps surae muscle, respectively. Higher stiffness may reflect a greater degree of severity of fibrosis and worse tissue repair, indicating that SWE examination might be an effective evaluation method for monitoring prognosis. Moreover, it is well-accepted that angiogenesis and reinnervation, arborization and growth are intimately connected processes. For example, in a model of transplanted human skin equivalents, Ferretti et al.^47^ found that vascularization precedes the process of innervation, indicating that the development of nerve tissue is driven by neurotrophic factors supplied by the vascular system. At present, few studies have been reported on the use of CEUS in evaluating angiogenesis in nerve regeneration. Therefore, we attempted to use CEUS examination to evaluate microblood perfusion of the regenerative nerve. In our CEUS examination, 450%PRP and 650%PRP therapies significantly improved the microblood perfusion of the regenerative nerve, as reflected by the PI and AUC values, and were positively correlated with VEGF expression. These two treatment groups also showed the best nerve regeneration, consistent with previous studies on vascular regrowth in animal models of sciatic nerve crush injury^48,49^. The vascular response to crush injury consists of two phases. The early phase, corresponding to the first week after injury, is characterized by an increase in blood vessel size accompanied by a constant number of blood vessels. Then, during the second phase, from 1 to 6 weeks after injury, an increase in the number of blood vessels is observed. The first phase of the vascular response is associated with the aggregation of macrophages and the removal of degenerated axons and myelin sheath fragments due to Wallerian degeneration, while the second phase involves nerve regeneration, which is composed of cellular proliferation, axonal elongation and myelination. Hence, revascularization plays an important role in peripheral nerve regeneration, and CEUS examination can also provide useful microcirculation perfusion information for assessing the prognosis of PNI. Comprehensive evaluation of the multimodality ultrasound and electrophysiological results revealed that the PRP-4.5× group and the PRP-6.5× group recovered well, while CMAP recovery was also better in these two groups than in the other treatment groups, which confirmed the usefulness of multimodality ultrasound in the evaluation of prognosis of peripheral nerve injury. Therefore, multimodality ultrasound, as a convenient and noinvasive detection method, may become a promising multidimensional imaging evaluation method for assessing the process of nerve crush injury and prognosis.

This study has several limitations. First, for platelet concentrates, in addition to variability in platelet content, the ‘ideal’ leukocyte concentration for different clinical conditions has yet to be elucidated in the literature. Second, since SCs play such a critical role in nerve regeneration, it would have been more meaningful to induce an analysis of the repair time together with electrophysiological and histological evaluations every 2 weeks. Third, high concentrations of PRP inhibited SC proliferation in vitro but did not significantly inhibit tissue regeneration in vivo. The reasons explaining these findings require further study and analysis. Finally, peripheral nerve ultrasound examination is conditioned by a number of difficulties, such as the size of the injured nerve, the depth of the lesion and the proficiency of the operator, as an experienced radiologist is needed to reliably examine results.

In conclusion, PRP, at the optimal concentration plays an active role in promoting the proliferation and neurotrophic function of SCs in vitro in a dose-dependent manner. Electrophysiological, histomorphometric and targeted muscle evaluation data revealed that serial ultrasound-guided PRP injections of PRP at an appropriate concentration for peripheral nerve crush injury accelerated the recovery of axonal function and dampened the atrophy of the target muscle. Moreover, multimodality ultrasound may provide a clinical reference for prognosis by allowing the stiffness and microcirculation perfusion of crush-injured peripheral nerves to be quantitatively evaluated.

## Methods

### Study design

**Fig. 9:**
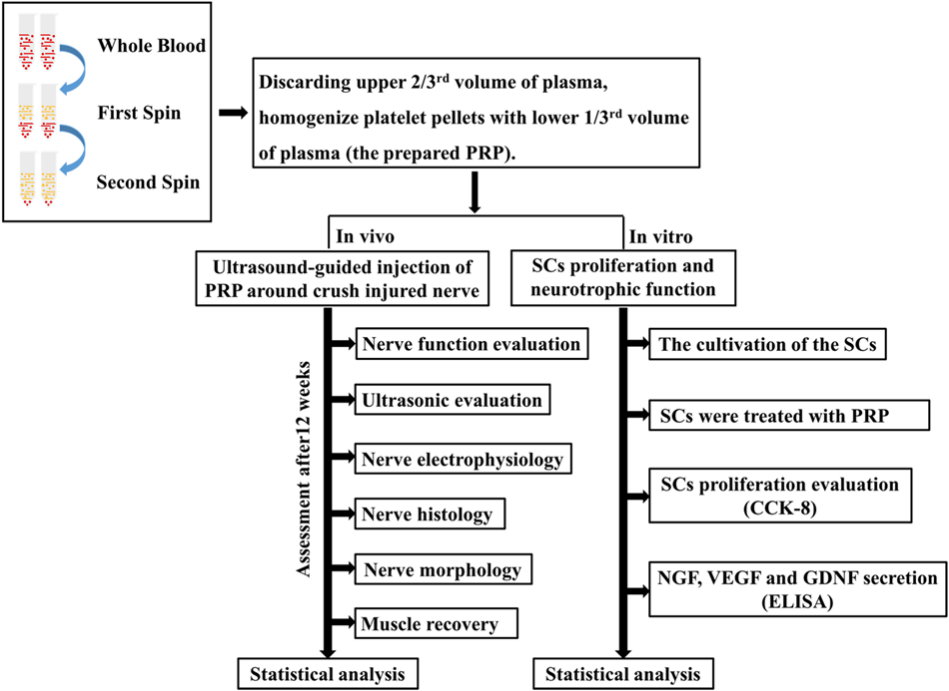
Illustration of the comprehensive steps in vitro and in vivo study. In vitro experiments mainly include the evaluation of the SCs proliferation and neurotrophic function treated with various concentrations of PRP. In vitro studies were conducted to evaluate the changes of ultrasonography, electrophysiology and histology of peripheral nerve crush injury treated with different concentrations of PRP.

The study design includes in vitro and in vivo studies (Fig. 9). This study was approved by the Ethical Committee of Experimental Animals of Chinese PLA General Hospital, Beijing, China (2015-x10–02). All experiments were performed at the Beijing Key Lab of Regenerative Medicine in Orthopaedics, Chinese PLA General Hospital, Beijing, China. All procedures described in this study were performed in accordance with the US National Institutes of Health Guide for the Care and Use of Laboratory Animals.

### Preparation of PRP

PRP was prepared via a modified method reported by Yamaguchi et al.^50^. A total of 5 ml whole blood was obtained through cardiac puncture of an anaesthetized 400 g adult Sprague–Dawley rat (Vital River Laboratory Animal Technology Co., Ltd., Beijing, China; license number SCXK (Jing) 2015–0001) and withdrawn into a 5 ml tube containing 3.8% w/v sodium citrate. The whole blood was immediately centrifuged at 400×*g* for 10 min and separated into three layers: a buffy coat (platelets), which is the most important, in the middle layer, acellular plasma in the supernatant at the top, and erythrocytes at the bottom. The upper layer and platelets were transferred to another non-anticoagulant tube and re-centrifuged at 800×*g* for 10 min. This second round of centrifugation separated the blood into two layers: acellular plasma at the top and PRP at the bottom. After discarding approximately three-quarters of the acellular plasma, the remainder (approximately 0.8 ml PRP) was extracted from the bottom of the tube. The number of platelets, erythrocytes and leukocytes from the prepared PRP and the whole blood of the rat was counted with an automated cell counter.

Different platelet concentrations were obtained relative to the concentrated platelet count (2130.41 ± 425.80 × 10^3^ platelets/ul, representing an increase of 6.5 times over the baseline venous count) by diluting with Dulbecco’s modified Eagle’s medium/nutrient mixture F-12 (DMEM/F-12; Gibco, USA). Platelet concentrations of 150%, 250%, 450%, and 650% in the whole blood counts were achieved. PRP of different concentrations was activated with a mixture of 10% calcium chloride (Sigma, c1016) solution and 5000 units of bovine thrombin (Sigma, T4648) to form PRP clots. The clots were then allowed to retract for 15 min. Next, the released supernatants with growth factors were isolated from the different aggregated platelets by centrifugation at 2800 × *g* for 10 min. The supernatants were stored in a −80 °C freezer until being used to supplement the culture medium or being assayed for growth factor concentration.

### Nerve cell culture

Schwann cells (SCs) were harvested and purified from the sciatic nerves of 72-hour-old Sprague–Dawley rats using previously established procedures^51^. Briefly, sciatic nerves were excised and minced from 10 Sprague–Dawley rats (Vital River Laboratory Animal Technology Co., Ltd., Beijing, China; license number SCXK (Jing) 2015–0001) and enzymatically dissociated with 1 ml of 0.2% (w/v) collagenase NB4 (Sigma-Aldrich) for 5 min. Next, the mixtures were placed on a magnetic stirrer at 37 °C for 10 min, centrifuged and resuspended in DMEM/F-12 (Gibco, USA) containing 10% (v/v) fetal bovine serum (FBS; Gibco), 1% penicillin–streptomycin solution (Gibco), 10 ng/ml heregulin-β1 (Sigma-Aldrich) and 2 mM forskolin (Sigma-Aldrich) in a 25 cm^2^ cell culture flask in an atmosphere of 5% CO_2_ at 37 °C. First passage (P2) cells were utilized in this study.

The SCs were identified by rabbit anti-S-100 immunostaining antibody (1:200, S2644, Sigma) prior to subsequent experiments. The SCs were diluted to a 1 × 10^6^/ml suspension, and 200 μl of the cell suspension was seeded in each hole of two 6-well culture plates, maintained with serum DMEM/F-12 medium for 24 h and allowed to attach overnight. Subsequently, the cells were grown for 3 days and 5 days following supplementation of 100 μl of five different supernatant preparations: (a) DMEM only (negative control), (b) 150% (PRP-1.5×, 480 × 10^3^ platelets/μl), (c) 250% (PRP-2.5×, 800 × 10^3^ platelets/μl), (d) 450% (PRP-4.5×, 1440 × 10^3^ platelets/μl), and (e) 650% (PRP-6.5×, 2081 × 10^3^ platelets/μl). After 3 and 5 days of SC culture, each well was supplemented with 300 μl 0.2% (w/v) collagenase NB4 (Sigma-Aldrich) for 1 min. The enzymatic reaction was terminated by adding DMEM/F-12 mixture containing 10% FBS, and the cell count was performed.

### SC proliferation

The initial number of cells in the culture medium was the same across the different treatment groups. SCs (2000 cells/well) were seeded in 96-well plates, and 50 μl of different supernatant preparations was added to each well in triplicate. At 1, 3, 5, and 7 days following the SC culture, a CCK-8 colorimetric assay (Cell Counting Kit-8; Dojindo Laboratories, Shanghai) was performed to evaluate the effect of PRP on the proliferation of SCs. The optical density was measured at 450 nm by using a microplate reader (Epoch, Biotek, US). The absorbance was directly proportional to the number of living cells.

### Measurement of NGF-β, VEGF, and GDNF secretion by ELISA

After 5 days of treatment with the indicated PRP, the culture medium was collected, and the concentrations of NGF-β, VEGF, and GDNF in the culture medium were determined using an NGF-β ELISA kit (Boster, EK0471), a VEGF ELISA kit (Boster, EK0540) and a GDNF ELISA kit (EK0363) as instructed by the manufacturer.

### Animals

Ninety 4-month-old healthy, male, clean New Zealand white rabbits with a mean weight of 2.5–3.0 kg were provided by the Animal Breeding Centre of Long’ an, Beijing, China (licence no. SCXK [Jing] 2014–0003). The animals were housed individually in cages in a quiet room under a 12-h light/dark cycle (lights on from 07:00 a.m. to 19:00 p.m.) at 22–24 °C ambient temperature. They were provided free access to water and standard rabbit nutrients.

The rabbits were randomly divided into five groups of 18 rabbits each. During the study, eleven animals died (some during the induction of anesthesia, some immediately postoperatively, and others due to diarrhea during feeding). The remaining rabbits included (1) a control group (*n* = 17) in which the sciatic nerve was exposed for 10 min followed by suturing the wound without crushing the nerve; (2) a model group (*n* = 15), which received sciatic nerve crush injury without any treatment; (3) a PRP-2.5× group (*n* = 15), which received sciatic nerve crush injury and treatment with PRP at a platelet concentration 2.5-fold greater than in whole blood; (4) PRP-4.5× group (*n* = 16), which received sciatic nerve crush injury and treatment with PRP at a platelet concentration 4.5-fold greater than in whole blood and (5) a PRP-6.5× group (*n* = 16), which received sciatic nerve crush injury and treatment with PRP at a platelet concentration 6.5-fold greater than in whole blood. In order to ensure the same number of animals in each group, 15 animals in each group were randomly selected for the following evaluation. For different statistical analyses (e.g., electrophysiology, ultrasound, and histological evaluation), 15 animals in each group were then randomly divided into 3 groups of 5 animals in each group.

### Surgical protocol

Animals were weighed and anaesthetized with an intramuscular injection of 3% pentobarbital sodium solution (1 ml/kg). All the animals had their hair removed from the dorsum of the waist to the right knee and were disinfected with iodine solution. All surgical procedures were performed under aseptic operating conditions by two surgeons. The right sciatic nerve was exposed from the sciatic notch to the level of popliteal branching using an intermuscular approach between the vastus lateralis and the biceps femoris muscles and was freed from the surrounding tissues via blunt dissection. After exposing the sciatic nerve, a 10-mm crush injury was induced using a needle holder (5-mm width) that was tightened to the third locking flange (held in place for 30 s) at 1 cm and 1.5 cm below the junction of the sciatic nerve and its small branches, and a piece of sterile catheter, as a mark of ultrasound detection, was fixed at the adjacent muscle near the junction site. The crushed area was translucent but not broken (Sunderland, grade III, axons and the endoneurium were damaged, but not the perineurium)^52^. Then, the muscule layer and skin were sutured with a 3–0 monofilament nylon suture. In the control group, the right sciatic nerve was exposed for 10 min, and the incision was closed.

### Postoperative care

Postsurgical infection was controlled by injection of antibiotics (800,000 IU of penicillin daily) intramuscularly for 5 days. The animals were monitored by a specialist veterinarian under standard laboratory conditions during the 3-month postoperative period.

### Autologous PRP preparation and injection

**Fig. 10:**
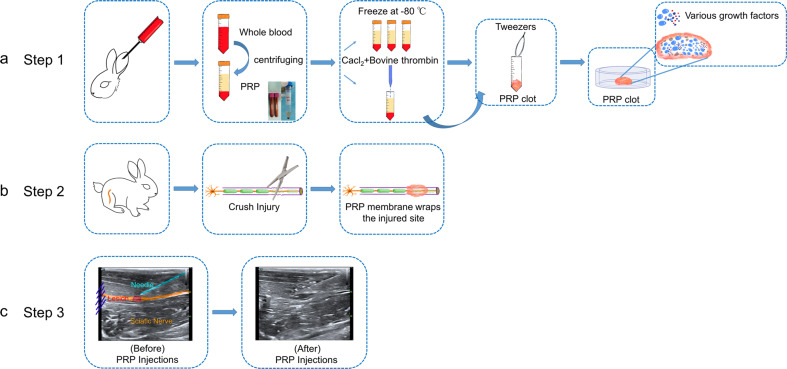
Schematic illustrations of the various steps of the experimental processes in vivo. **a** Flowchart describing the preparation of PRP and PRP clot. **b** Establishment of the crush injury model and PRP treatment process during the operation, including the needle holder that crushed the right sciatic nerve and the intraoperative PRP membrane that immediately wrapped the nerve lesion. **c** Ultrasound-guided post-operative PRP injections. PRP platelet-rich plasma.

Before surgery, a total of 25 ml of whole blood was extracted from the central ear artery of each rabbit in the crush injury groups and withdrawn into tubes containing 3.8% w/v sodium citrate. The blood was first centrifuged at 400×*g* for 10 min at 22 °C and separated into three layers. The acellular plasma in the top layer and the platelets in the middle layer were then transferred to another sterile tube without anticoagulant followed by a second round of centrifugation at 800×*g* for 10 min. After discarding the upper 2/3 of the platelet-poor plasma, 5 ml of PRP was pipetted from the bottom of the tube. For quality testing, an automated hemocytometer was used to count the number of red blood cells, white blood cells and platelets in 250 μl whole blood and 250 μl PRP. According to the results of in vitro experiments, the three platelet concentrations were selected (PRP-2.5×, PRP-4.5×, PRP-6.5×) for testing repair of sciatic nerve crush injury in vivo. Namely, the platelet concentration in the PRP-2.5× group was 2.5 times the PRP concentration in whole blood; similarly, the platelet concentrations in the PRP-4.5× and PRP-6.5× groups were 4.5 times and 6.5 times that in whole blood, respectively (Fig. 10a).

After that, PRP membranes with different concentrations of PRP according to each group were used to wrap the injured site of the nerve, and then, serial injections of activated PRP were administered under ultrasound guidance. Briefly, 1 ml diluted PRP was extracted and activated with a 10% calcium chloride (Sigma, c1016) solution and 5000 units of bovine thrombin (Sigma, T4648) to form a PRP gel. Immediately after inducing the crush injury to the sciatic nerve in the PRP-2.5×, PRP-4.5×, and PRP-6.5× groups, the nerve lesion was wrapped with a PRP membrane (Fig. 10b). A total of 6 ml of the diluted PRP was divided into 3 equal portions and stored in a −80 °C freezer. Prior to injection, the PRP was mixed with 10% calcium chloride (10:1, Sigma, c1016) to activate the platelets.

All of the ultrasound procedures were performed by a radiologist with nine years of ultrasound experience. Ultrasound-guided PRP injections were administered in the PRP-2.5× group, the PRP-4.5× group and the PRP-6.5× group using a 4–15 MHz linear-array probe (Mindray, Resona 7, Fig. 10c) at a volume of 2 ml per injection at 2, 4, and 6 weeks. An 18-G needle was used and placed near the epineurium, and 2 ml of PRP was injected and delivered to lift the nerve off the upper tissue and the underlying tissue (Fig. 10c). After the injection, the entire sciatic nerve was detected using high-frequency ultrasound, which helps ensure that the PRP had spread from the proximal region to the distal region of the lesion site.

### Neurological function evaluation

The sciatic nerve was evaluated at 8 (*n* = 15 animals/group) and 12 weeks (*n* = 15 animals/group). The toe spreading score (graded from 1 to 4 points)^53^ and the modified Tarlov score (rated from 0 to 4 points)^54^ were summed together to obtain the total neurological function score. Higher scores indicated better sciatic nerve function.

### Nerve multimodality ultrasound evaluation

The thickness, stiffness and perfusion of the sciatic nerve (*n* = 5/ each group) were examined at 12 weeks. Briefly, following the induction of anesthesia, the sciatic nerve was scanned longitudinally, and the damaged area was identified by a catheter near the epineurium. The thickness of the injured nerve was measured for 5 times using high-frequency ultrasonic equipment (Vevo 3100, Visualsonics, Canada) with a 14-MHz to 28-MHz linear array probe (MX250); the stiffness of the damaged site was measured using an Aixplorer ultrasound system (Supersonic Imagine, Aixen-Provence, array transducer Super Linear L10–2, France) equipped with SWE, and the size of the circular region of interest (ROI) was fixed to 1 mm in all sciatic nerves. The mean Young’s modulus was expressed and averaged for 5 measurements. Then, CEUS imaging, a method for detecting the microcirculation of the sciatic nerve, was performed using a high-resolution ultrasound system (Mindray, Resona 7) equipped with a linear array transducer (4–15 MHz) at a low mechanical index (MI 0.05–0.07). SonoVue (Bracco International, Milan, Italy) is a sulfur-hexafluoride-filled microbubble contrast agent encapsulated by a flexible phospholipid shell. Individual microbubbles (2–5 μm) are similar in size to red blood cells, resulting in the agent remaining solely in the intravascular compartment, and almost all the gas is exhaled by respiration within 15 min of intravenous injection. SonoVue was injected into a peripheral ear vein at a bolus of 0.13 ml/kg, followed by a 2 ml saline flush for the CEUS examination. The contrast-enhancement process and the timer on the US machine were started, and then, real-time dynamic images of at least 60 s in length were digitally stored. The area under the curve (AUC) indicated the relative blood volume in the repaired tissue, and the peak intensity (PI) corresponded to the maximal signal intensity at a single injection of contrast agent. Higher AUC and PI indicate the better blood perfusion. The parameters are presented for 5 measurements.

### Electrophysiological recovery analysis

After ultrasound detection, electrophysiological tests were performed at 12 weeks (*n* = 5 animals/group). The sciatic nerve on the right side was re-exposed under anesthesia and stimulated by two electrodes (10 Hz, 6 mA) that were placed at the proximal and distal ends of the regenerated nerve stumps. The compound muscle action potential (CMAP) amplitudes and latencies were recorded 5 times on the targeted triceps surae muscle.

### Histological evaluation of regenerated nerves

At 12 weeks, another 5 animals from each group were randomly selected to harvest regenerated nerve tissues for histological assessment. To determine the percentage of the blue-stained collagen area, the nerve tissues were fixed using 4% paraformaldehyde for 24 h and embedded in paraffin, and 4 µm longitudinal sections were obtained for Masson’s trichrome staining. Five visual fields were randomly selected from each sample, and the percentage of collagen area was measured with Image-Pro Plus (IPP) 6.0 software (×20 magnification, Media Cybernetics, Inc., Rockville, MD, USA)

The tissues that were used for Masson’s trichrome staining were also used for immunofluorescence staining (5 animals/group). The 4 µm longitudinal sections were washed in PBS and incubated at 4 °C overnight with mouse anti-Neurofilament 200 antibody (1:200, N5389, Sigma) and mouse anti-VEGF antibody (1:100, MA5–13182, Pierce, US). The next morning, the samples were washed with PBS three times and immediately incubated with the secondary antibodies (goat anti-mouse IgG, diluted to 1:200 in PBS) conjugated with Alexa Fluor 594 for 2 h at room temperature. After 3 washes with PBS for 5 min each, the sections were stained with DAPI (4’,6-dia-mino-2-phenylindole) for 10 min. All sections were observed with a fluorescence microscope (×200 magnification, Nikon Eclipse C1, Japan), and the recovery of the nerve fiber was quantitatively compared among groups by integrated optical density (IOD) analysis.

### Histomorphometrical assessment of regenerated nerves

At 12 weeks, 1.5-µm thick semithin sections and 70-nm thick ultrathin sections were acquired from the 5 mm distal region of the injured nerve segments from the remaining animals (*n* = 5 animals/group). IPP software was used to calculate the density of myelinated nerve fibers, fiber diameter and myelin sheath thickness. Five randomly selected visual fields were selected for each sample in each group.

### Triceps surae muscle ultrasound evaluation

High-frequency ultrasound equipment with a 21-MHz to 44-MHz linear array probe (MX4000, Vevo 3100, Visualsonics, Canada) was used for conventional ultrasound assessment of muscle (*n* = 5 animals/group). The echogenicity of the triceps surae muscle was observed longitudinally, and the thickness of targeted muscle was measured transversely to show the maximal cross-sectional area (CSA) of the muscle. In addition, the stiffness of the triceps surae muscle was measured with an Aixplorer ultrasound system (Supersonic Imagine, Aixen-Provence, array transducer Super Linear L10–2, France) equipped with SWE (*n* = 5 animals/group). The ROI size was fixed 10–11 mm in all cases. Care was taken not to place the circular ROIs near the bone. Five measurements were taken in each case for the statistical analysis.

### Triceps surae muscle wet weight ratio and morphological evaluation

The animals were sacrificed at the end of 12 weeks after the functional tests, and ultrasound and electrophysiological examinations were completed (*n* = 5 animals/group). The triceps surae muscles were harvested and weighed to calculate the muscle wet weight ratio (ipsilateral/contralateral side). To determine the percentage of collagen area and the average CSA of muscle fibers, the same procedure used for Masson’s trichrome staining of the nerve was employed. Five random visual fields (200× magnification) were selected in each sample, and the IPP software was used for quantitative analysis.

### Statistical analysis

Statistical analyses were performed using GraphPad Prism 8.0 (GraphPad Software, Inc. San Diego, CA, United States) and Statistical Program for Social Sciences (SPSS) software (version 22.0). The Kolmogorov-Smirnov test was used to test for a normal distribution of the data. Student’s t-test was used to compare differences between two groups and one-way ANOVA was used for multiple comparisons. Tukey’s multiple comparison post hoc test was applied when *P* > 0.05 in the test of homogeneity of variances; otherwise, Dunnett’s T3 post hoc test was applied. Pearson’s correlation analysis was performed between ultrasonic variables and histopathological parameters. *P* < 0.05 between groups was considered statistically significant. According to the post hoc power analysis, a power >80% was obtained in each statistical analysis at 12 weeks at a significance level of 0.05. No additional animals were needed.

### Reporting summary

Further information on experimental design is available in the Nature Research Reporting Summary linked to this paper.

## Supplementary information

Reporting Summary Checklist FLAT

## Data Availability

The data sets generated during and/or analyzed during this study are available from the corresponding author on reasonable request.
