# Supplementary material for: Ultrasound-guided platelet-rich plasma injection and multimodality ultrasound examination of peripheral nerve crush injury
Source: NPJ Regen Med. 2020 Nov 20;5:21. doi: 10.1038/s41536-020-00101-3 (PMC7680141; doi:10.1038/s41536-020-00101-3)
Supplement: Supplementary file 1 — Reporting Summary Checklist FLAT [file 41536_2020_101_MOESM1_ESM.pdf]

nature research

Corresponding author(s): Yukun Luo; Yuexiang Wang

Last updated by author(s): Yukun Luo; Yuexiang Wang

Reporting Summary

Nature Research wishes to improve the reproducibility of the work that we publish. This form provides structure for consistency and transparency in reporting. For further information on Nature Research policies, see our Editorial Policies and the Editorial Policy Checklist.

Statistics

For all statistical analyses, confirm that the following items are present in the figure legend, table legend, main text, or Methods section.

n/a	Confirmed	
☐	☒	The exact sample size (n) for each experimental group/condition, given as a discrete number and unit of measurement
☐	☒	A statement on whether measurements were taken from distinct samples or whether the same sample was measured repeatedly
☐	☒	The statistical test(s) used AND whether they are one- or two-sided<br>*Only common tests should be described solely by name; describe more complex techniques in the Methods section.*
☐	☒	A description of all covariates tested
☐	☒	A description of any assumptions or corrections, such as tests of normality and adjustment for multiple comparisons
☐	☒	A full description of the statistical parameters including central tendency (e.g. means) or other basic estimates (e.g. regression coefficient) AND variation (e.g. standard deviation) or associated estimates of uncertainty (e.g. confidence intervals)
☐	☒	For null hypothesis testing, the test statistic (e.g. F, t, r) with confidence intervals, effect sizes, degrees of freedom and P value noted<br>*Give P values as exact values whenever suitable.*
☒	☐	For Bayesian analysis, information on the choice of priors and Markov chain Monte Carlo settings
☒	☐	For hierarchical and complex designs, identification of the appropriate level for tests and full reporting of outcomes
☐	☒	Estimates of effect sizes (e.g. Cohen's d, Pearson's r), indicating how they were calculated

Our web collection on statistics for biologists contains articles on many of the points above.

Software and code

Policy information about availability of computer code

Data collection	GraphPad Prism 8.0 (GraphPad Software, Inc. San Diego, CA, United States) and Statistical Program for Social Sciences (SPSS) software (version 22.0).
Data analysis	GraphPad Prism 8.0 (GraphPad Software, Inc. San Diego, CA, United States) and Statistical Program for Social Sciences (SPSS) software (version 22.0).

For manuscripts utilizing custom algorithms or software that are central to the research but not yet described in published literature, software must be made available to editors and reviewers. We strongly encourage code deposition in a community repository (e.g. GitHub). See the Nature Research guidelines for submitting code & software for further information.

Data

Policy information about availability of data

All manuscripts must include a data availability statement. This statement should provide the following information, where applicable:
- Accession codes, unique identifiers, or web links for publicly available datasets
- A list of figures that have associated raw data
- A description of any restrictions on data availability

The data sets generated during and/or analysed during the current study are available from the corresponding author on reasonable request.

Field-specific reporting

Please select the one below that is the best fit for your research. If you are not sure, read the appropriate sections before making your selection.

☒ Life sciences ☐ Behavioural & social sciences ☐ Ecological, evolutionary & environmental sciences

For a reference copy of the document with all sections, see nature.com/documents/nr-reporting-summary-flat.pdf

Life sciences study design

All studies must disclose on these points even when the disclosure is negative.

Sample size	Twelve weeks after surgery, the online website ClinCalc.com was used to verify whether each group had enough samples. According to the main experimental results, PRP-4.5x group significantly promoted nerve repair compared with PRP-2.5x group, and the results of main evaluation indexes (electrophysiological results, ultrasound results and immunofluorescence results) were selected to calculate the sample size. Therefore, using independent-sample study analysis of variance as a precondition (α err rate=0.05; β err rate =0.2; Power=0.8; Number of groups=2), with the following setups: For example, based on the CMAP amplitude of PRP-4.5x group & PRP-2.5x group (27.40±3.07 mV vs. 22.21±2.81 mV), the sample size was 5 in each group. Moreover, through a post hoc power analysis, a power of 80% (n=5 in each group) was obtained at a significance level of 0.05. So no more animals needed. The method of calculating sample size based on other main indicators was similar to the above method.To sum up, the sample size of the groups with different evaluation methods calculated based on the final results was all less than or equal to 5, so no more animals needed.
Data exclusions	During the study, eleven animals died (some during the induction of anesthesia, some immediately postoperatively, and others due to diarrhea during feeding)
Replication	Our results showed that the PRP with a 4.5-fold concentration of whole blood platelets could significantly stimulate the proliferation and secretion of SCs and nerve repair. The PRP-6.5x group showed similar nerve repair as the PRP-4.5x group in vivo at 12 weeks after crush injury. In addition, the changes in stiffness and blood perfusion were positively correlated with the collagen area percentage and VEGF expression in the injured nerve, respectively. Thus, serial ultrasound-guided PRP injections of PRP at an appropriate concentration for peripheral nerve crush injury accelerated the recovery of axonal function and dampened the atrophy of the target muscle. Moreover, multimodality ultrasound techniques provide a clinical reference for prognosis by allowing the stiffness and microcirculation perfusion of crush-injured peripheral nerves to be quantitatively evaluated.
Randomization	In order to ensure the same number of animals in each group, 15 animals in each group were randomly selected for the following evaluation. For different statistical analyses (e.g., electrophysiology, ultrasound, and histological evaluation), 15 animals in each group were then randomly divided into 3 groups of 5 animals in each group.
Blinding	All of the ultrasound procedures were performed by a radiologist in blind method with nine years of ultrasound experience.

Reporting for specific materials, systems and methods

We require information from authors about some types of materials, experimental systems and methods used in many studies. Here, indicate whether each material, system or method listed is relevant to your study. If you are not sure if a list item applies to your research, read the appropriate section before selecting a response.

Materials & experimental systems

n/a	Involved in the study	
☐	☐	Antibodies
☐	☐	Eukaryotic cell lines
☐	☐	Palaeontology and archaeology
☐	☒	Animals and other organisms
☐	☐	Human research participants
☐	☐	Clinical data
☐	☐	Dual use research of concern

Methods

n/a	Involved in the study	
☒	☐	ChIP-seq
☒	☐	Flow cytometry
☒	☐	MRI-based neuroimaging

Antibodies

Antibodies used	mouse anti-Neurofilament 200 antibody (1:200, N5389, Sigma); mouse anti-VEGF antibody (1:100, MA5-13182, Pierce, US);rabbit anti-S-100 immunostaining antibody （1:200, S2644, Sigma）
Validation	N5389: Monoclonal Anti-Neurofilament 200 (mouse IgG1 isotype) is derived from the hybridoma produced by the fusion of mouse myeloma cells and splenocytes from an immunized mouse. The neurofilaments are one of the five major groups of intermediate

filaments (IFs) and are found predominantly in cells or tissues of neuronal origin. They are composed of three major proteins of apparent molecular weights 68 kD, 160 kD, and 200 kD and are named as NEFL (light), NEFM (medium) and NEFH (heavy) respectively.[1] Neurofilament proteins are synthesized in the neuronal perikarya, assembled to form filaments and then slowly transported within the axons towards the synaptic terminals.
MA5-13182: VEGF (vascular endothelial growth factor) which is a 45 kDa homodimeric, disulfide-linked glycoprotein involved in angiogenesis which promotes tumor progression and metastasis. VEGF has a variety of effects on vascular endothelium, including the ability to promote endothelial cell viability, mitogenesis, chemotaxis, and vascular permeability. The VEGF family currently includes VEGF-A, VEGF-B, VEGF-C, VEGF-D, VEGF-E, and PIGF. VEGF and its receptor system have been shown to be the fundamental regulators in the cell signaling of angiogenesis. Most tumors have the absolute requirement of angiogenesis, and VEGF has been described as the most potent angiogenic cytokine linked to this process. To date 5 different isoforms of VEGF have been described. These isoforms are generated as the result of alternative splicing from a single VEGF gene. These various isoforms have been shown to bind to two tyrosine-kinase receptors flt-1 (VEGFR-1) and flk-1/KDR (VEGFR-2), which have been found to be expressed almost exclusively on endothelial cells. VEGF and its high-affinity binding receptors, the tyrosine kinases FLK1 and FLT1, are thought to be important for the development of embryonic vasculature. Studies have shown that an alternately spliced form of FLT1 produces a soluble protein, termed sFLT1, which binds vascular endothelial growth factor with high affinity, playing an inhibitory role in angiogenesis. Elevated levels of VEGF is linked to POEMS syndrome (Polyneuropathy, Organomegaly, Endocrinopathy, Monoclonal gammopathy, Skin changes) also known as Crow-Fukase syndrome which affects multiple organs in the body.
S2644: Antibodies against Proteins/Bioactives/Markers/Receptors for Stem Cell Biology, Antibodies for Neural Stem Cells, Antibodies for Stem Cell Biology, Antibodies to Actin-associated Proteins/Myosin, General Stem Cell Biology, Glial Markers, Neural Stem Cell Antibodies and Tracking, Neural Stem Cell Biology, S1-SD, Stem Cell Characterization.

Eukaryotic cell lines

Policy information about cell lines

Cell line source(s) — *State the source of each cell line used.*

Authentication — *Describe the authentication procedures for each cell line used OR declare that none of the cell lines used were authenticated.*

Mycoplasma contamination — *Confirm that all cell lines tested negative for mycoplasma contamination OR describe the results of the testing for mycoplasma contamination OR declare that the cell lines were not tested for mycoplasma contamination.*

Commonly misidentified lines (See ICLAC register) — *Name any commonly misidentified cell lines used in the study and provide a rationale for their use.*

Palaeontology and Archaeology

Specimen provenance — *Provide provenance information for specimens and describe permits that were obtained for the work (including the name of the issuing authority, the date of issue, and any identifying information).*

Specimen deposition — *Indicate where the specimens have been deposited to permit free access by other researchers.*

Dating methods — *If new dates are provided, describe how they were obtained (e.g. collection, storage, sample pretreatment and measurement), where they were obtained (i.e. lab name), the calibration program and the protocol for quality assurance OR state that no new dates are provided.*

☐ Tick this box to confirm that the raw and calibrated dates are available in the paper or in Supplementary Information.

Ethics oversight — *Identify the organization(s) that approved or provided guidance on the study protocol, OR state that no ethical approval or guidance was required and explain why not.*

Note that full information on the approval of the study protocol must also be provided in the manuscript.

Animals and other organisms

Policy information about studies involving animals; ARRIVE guidelines recommended for reporting animal research

Laboratory animals — 4-month-old healthy, male, clean New Zealand white rabbits with a mean weight of 2.5–3.0 kg

Wild animals — The study did not involve wild animals.

Field-collected samples — The study did not involve samples collected from fields.

Ethics oversight — This study was approved by the Ethical Committee of Experimental Animals of Chinese PLA General Hospital, Beijing, China (2015-x10-02).

Note that full information on the approval of the study protocol must also be provided in the manuscript.

Human research participants

Policy information about studies involving human research participants

Population characteristics	*Describe the covariate-relevant population characteristics of the human research participants (e.g. age, gender, genotypic information, past and current diagnosis and treatment categories). If you filled out the behavioural & social sciences study design questions and have nothing to add here, write "See above."*
Recruitment	*Describe how participants were recruited. Outline any potential self-selection bias or other biases that may be present and how these are likely to impact results.*
Ethics oversight	*Identify the organization(s) that approved the study protocol.*

Note that full information on the approval of the study protocol must also be provided in the manuscript.

Clinical data

Policy information about clinical studies

All manuscripts should comply with the ICMJE guidelines for publication of clinical research and a completed CONSORT checklist must be included with all submissions.

Clinical trial registration	*Provide the trial registration number from ClinicalTrials.gov or an equivalent agency.*
Study protocol	*Note where the full trial protocol can be accessed OR if not available, explain why.*
Data collection	*Describe the settings and locales of data collection, noting the time periods of recruitment and data collection.*
Outcomes	*Describe how you pre-defined primary and secondary outcome measures and how you assessed these measures.*

Dual use research of concern

Policy information about dual use research of concern

Hazards

Could the accidental, deliberate or reckless misuse of agents or technologies generated in the work, or the application of information presented in the manuscript, pose a threat to:

No	Yes	
☒	☐	Public health
☒	☐	National security
☒	☐	Crops and/or livestock
☒	☐	Ecosystems
☒	☐	Any other significant area

Experiments of concern

Does the work involve any of these experiments of concern:

No	Yes	
☒	☐	Demonstrate how to render a vaccine ineffective
☒	☐	Confer resistance to therapeutically useful antibiotics or antiviral agents
☒	☐	Enhance the virulence of a pathogen or render a nonpathogen virulent
☒	☐	Increase transmissibility of a pathogen
☒	☐	Alter the host range of a pathogen
☒	☐	Enable evasion of diagnostic/detection modalities
☒	☐	Enable the weaponization of a biological agent or toxin
☒	☐	Any other potentially harmful combination of experiments and agents
